# Editorial: Decoding the neuroanatomy of addiction: insights into substance use disorders

**DOI:** 10.3389/fnana.2026.1786383

**Published:** 2026-02-03

**Authors:** Darlene A. Mitrano, Janet D. Robishaw

**Affiliations:** 1 Department of Biology, Chemistry, and Environmental Science and Program in Neuroscience, Christopher Newport University, Newport News, VA, United States; 2 Department of Biomedical Science, Charles E. Schmidt College of Medicine, Florida Atlantic University, Boca Raton, FL, United States; 3 Department of Comparative, Diagnostic, and Population Medicine, College of Veterinary Medicine, University of Florida, Gainesville, FL, United States

**Keywords:** addiction, alcohol and stimulants, neuroanatomy, substance use disorders, treatments

## 1 Introduction

We are excited to share a series of articles centered on *Decoding the Neuroanatomy of Addiction: Insights into Substance Use Disorders*. Across six manuscripts, we present perspectives on different drugs of abuse, all with a neuroanatomical basis. These studies span the molecular level of examining the role of the heterotrimeric G-protein complex components (Pelletier et al.), to the organismal level, assessing substance use disorder treatments and outcomes in human patients (Galiza Soares et al.; Calleja-Conde et al.; Liu et al.).

Why do we continue to examine the causes and effects of substance use disorders, treatment, and relapse? In the United States, one in four individuals over age 12 reported using illicit drugs in 2024, and more than 134 million had used alcohol in the past month [Substance Abuse and Mental Health Services Administration (SAMHSA), 2025]. Of those surveyed, 16.8 percent, about 48 million people, met criteria for a substance use disorder [Substance Abuse and Mental Health Services Administration (SAMHSA), 2025]. According to the DSM-5, SUDs involve recurrent and compulsive use of alcohol and, or drugs, that lead to health problems and impaired responsibilities at home, school, or work (American Psychiatric Association, 2013). These numbers highlight that SUDs remain a major public health issue requiring continued scientific attention.

## 2 Advances at the molecular, receptor, and circuit level, stimulants and alcohol

Cocaine, methamphetamine, and prescription stimulants, continue to present challenges, with an estimated nine million Americans misusing stimulants in 2024 [Substance Abuse and Mental Health Services Administration (SAMHSA), 2025]. Pelletier et al. examined two prominent GPCRs in the striatum, the dopamine D1 receptor and the adenosine 2A receptor, focusing on the G_olf_ β_2_γ_7_ complex. They showed that the *Gng7* gene encodes multiple γ_7_ subunit forms, with Transcript 1 most common in dendrites and spines. Loss of the G_olf_ protein and β_2_γ_7_ altered spine morphology and changed cocaine sensitization in genetically modified mice, identifying a potential molecular target for stimulant dependence treatment (Pelletier et al.).

Not all stimulants act typically. Modafinil, a wake-promoting drug, affects dopamine but does not exhibit the same addictive properties (Szabadi). Reinforcing and locomotor effects of stimulants are linked to projections from the VTA to nucleus accumbens and from the substantia nigra to the dorsal striatum. Wakefulness, on the other hand, appears to involve dopamine release from the substantia nigra, VTA, and the ventral periaqueductal gray, dorsal raphe to the prefrontal cortex, along with broader networks including the locus coeruleus, thalamus, and ventral pallidum (Szabadi). Distinguishing reinforcement from arousal may help clarify modafinil's mechanism and guide development of less addictive wake-promoting treatments.

Relapse often occurs when individuals encounter drug associated cues, such as familiar environments or stressors. Hauser et al. examined inhibitory cues, odors linked to extinction of alcohol self-administration, and excitatory cues, odors associated with alcohol access, to assess the involvement of serotonin 5-HT_7_ receptors in the nucleus accumbens shell. Using alcohol preferring rats, they showed that these opposing cues produced differential effects on dopamine, glutamate and serotonin levels. Activation of the 5-HT_7_ receptor reduced reinstatement triggered by excitatory cues, suggesting this receptor as a potential target for reducing alcohol consumption.

## 3 Effects of the COVID-19 pandemic on SUDs

COVID-19 had widespread effects on substance use. Many individuals increased their use of drugs or alcohol to cope with stress, emotional strain, and isolation (Abramson, 2021). Reports of loneliness rose during this period (O'Sullivan et al., 2021; Jabbari et al., 2024). Those with existing SUDs experienced poorer health outcomes and were more susceptible to infection (Abramson, 2021; Calleja-Conde et al.). At the same time, drug related overdose deaths exceeded 100,000 in 2021, with synthetic opioids contributing substantially (Spencer et al., 2022).

Galiza Soares et al. reviewed opioid use disorder susceptibility and withdrawal effects, comparing human findings with rodent models of juvenile social isolation, maternal separation, and limited bedding, nesting. They identified overlapping brain regions, including the nucleus accumbens, orbitofrontal cortex, VTA, and amygdala, implicated in both social isolation and opioid dependence (Galiza Soares et al.). The authors propose that altered neurotransmission, particularly within opioid systems, may weaken social attachment and heighten vulnerability to reinforcing drug effects (Galiza Soares et al.).

Calleja-Conde et al. reviewed the effects of COVID-19 infection in individuals with alcohol use disorder (AUD). Both COVID-19 and AUD independently disrupt immune function, contribute to neuroinflammation, and alter the gut-brain axis, leading to mental health consequences. Their review suggests a synergistic interaction between AUD and COVID-19, associated with increased rates of Long COVID and higher mortality (Calleja-Conde et al.).

## 4 Emerging therapies, exercise for SUD recovery

Traditional SUD treatment typically includes detoxification, behavioral therapy, and a pharmacological substitution. Due to high relapse rates, researchers are exploring complementary approaches. Liu et al., reviewed more than 30 publications examining aerobic and yogic exercise in human SUD treatment. They found that aerobic exercise correlates with improvements in physical health, potentially mediated by intracellular signaling pathways and anti-inflammatory factors (Liu et al.). Yoga and other mind body practices were associated with improved sleep quality and cognitive function, possibly through modulation of the HPA axis. These findings support incorporating exercise-based interventions as adjuncts or additions to traditional addiction treatment programs.

## 5 Summary and conclusions

This editorial and compilation of manuscripts advances the field of addiction neuroscience by integrating new molecular, circuit-level, and clinical findings that refine our understanding of substance use disorders and highlight emerging therapeutic targets. The issue identifies the G_olf_β_2_γ_7_ complex as a novel regulator of stimulant responses, clarifies dopamine pathways relevant to reinforcement vs. arousal, and demonstrates that 5-HT_7_ receptor signaling can modulate cue-induced alcohol seeking. It also synthesizes evidence showing how social isolation, immune dysregulation, and gut–brain axis disturbances interact with opioid and alcohol use vulnerability. Finally, the Research Topic strengthens the evidence base for exercise-based interventions by outlining biological mechanisms through which aerobic and mind–body practices may support recovery, offering a more mechanistic foundation for integrating these approaches into substance use disorder treatment.
